# How are mathematical models and results from mathematical models of vaccine-preventable diseases used, or not, by global health organisations?

**DOI:** 10.1136/bmjgh-2021-006827

**Published:** 2021-09-06

**Authors:** Paula Christen, Lesong Conteh

**Affiliations:** 1 Department of Infectious Disease Epidemiology, Imperial College London, London, UK; 2 Department of Health Policy, The London School of Economics and Political Science, London, UK

**Keywords:** immunisation, public health, vaccines, epidemiology

## Abstract

While epidemiological and economic evidence has the potential to provide answers to questions, guide complex programmes and inform resource allocation decisions, how this evidence is used by global health organisations who commission it and what organisational actions are generated from the evidence remains unclear. This study applies analytical tools from organisational science to understand how evidence produced by infectious disease epidemiologists and health economists is used by global health organisations. A conceptual framework that embraces evidence use typologies and relates findings to the organisational process of action generation informs and structures the research. Between March and September 2020, we conducted in-depth interviews with mathematical modellers (evidence producers) and employees at global health organisations, who are involved in decision-making processes (evidence consumers). We found that commissioned epidemiological and economic evidence is used to track progress and provides a measure of success, both in terms of health outcomes and the organisations’ mission. Global health organisations predominantly use this evidence to demonstrate accountability and solicit funding from external partners. We find common understanding and awareness across consumers and producers about the purposes and uses of these commissioned pieces of work and how they are distinct from more academic explorative research outputs. Conceptual evidence use best describes this process. Evidence is slowly integrated into organisational processes and is one of many influences on global health organisations’ actions. Relationships developed over time and trust guide the process, which may lead to quite a concentrated cluster of those producing and commissioning models. These findings raise several insights relevant to the literature of research utilisation in organisations and evidence-based management. The study extends our understanding of how evidence is used and which organisational actions are generated as a result of commissioning epidemiological and economic evidence.

**Key questions**

**What is already known?**

- Despite numerous initiatives to enhance knowledge utilisation, the understanding of how burden of disease and vaccine impact estimates are used by global health organisations that commission research evidence production is limited.

**What are the new findings?**

- Our study draws on an organisation science framework to examine the role of burden of disease and vaccine impact estimates for global health organisations.
- We found that burden of disease and vaccine impact estimates are not directly translated into organisational actions but are used by global health organisations to demonstrate accountability, to solicit funding and to get the buy-in from external partners.
- We found that evidence consumers can perceive evidence as ‘negative’, if their entrenched knowledge, built up over time, is challenged, or if the evidence does not meet their expectations, thus impeding its use.

**What do the new findings imply?**

- Interactions between evidence producers and consumers are vital so that evidence is produced in correspondence to questions of evidence consumers.
- Commissioned models, global in their scope, appear to serve a specific purpose, distinct from other academic modelling efforts.
- Their value lies in their ability to track progress and thus advocate for resources.
- Is there a missed opportunity given their disconnect from national-level resource allocation decisions?
- Cocreating a research agenda may enhance evidence consumers’ willingness to embrace model limitations and caveats thus allowing for more innovative methods to be employed in the evidence production process.

## Introduction

In addition to domestic efforts, global health organisations (GHOs), such as the Bill & Melinda Gates Foundation and associated partnerships, such as the Global Alliance for Vaccines and Immunization, play a prominent role in low and middle-income country (LMIC) immunisation programmes.^1^ Donor assistance for health (DAH) from global, bilateral and multilateral partnerships and agencies targeting immunisation totalled $34.5 billion between 1990 and 2016,^2^ contributing to impressive health gains.^3 4^ Nonetheless, inequalities in the provision of universal immunisation for all children, as initially set out by the WHO in 1974 through the Expanded Program on Immunization, continue to exist between and within countries.^5^ These inequalities are even more pronounced in terms of access to COVID-19 vaccinations.^6^

In the effort to act on the impact of long-standing, emerging and re-emerging infectious diseases, GHOs work closely together with researchers by commissioning research projects that have the potential to solve complex problems.^7–9^ Epidemiologists are commissioned by these organisations to estimate the impact of vaccines using mathematical models of infectious diseases. Epidemiological models can estimate the spread of infectious diseases in a given population and quantify the number of lives saved or deaths averted through public health interventions. Estimated number of deaths due to a disease can be used to inform other generic metrics (eg, disability-adjusted life years) to measure burden of disease and vaccine impact estimates (BOD & VIE) (eg, ref 10). The BOD was estimated under different vaccination coverage scenarios across 10 pathogens in 98 LMICs.^10^ This type of evidence has the potential to provide answers to policy questions,^11^ guide complex policy problems and inform resource allocation decisions.^12–14^

Despite numerous initiatives to enhance knowledge utilisation, the understanding of how BOD & VIE are used by GHOs is limited. Research utilisation studies have predominantly been conducted in other organisational contexts. Therefore, the objective of this study was to understand how BOD & VIE are used by the GHOs that commission the research and what actions are generated following the commissioning of evidence production.

## Methods

### Study design and sampling

Research for this paper was informed by a conceptual framework, which addresses possible types of evidence use throughout different organisational processes.^15^ The conceptual framework draws from organisation science, specifically Beyer and Trice’s literature review, which critically assessed the phenomenon of research utilisation in organisations systematically.^15^ We explicitly focus on the organisational process of *action generation*. Evidence use in the process of action generation is ascribed to three overarching categories of evidence use: instrumental, conceptual and symbolic.^16^ The typologies were initially proposed by Weiss^17^ and other pioneering scholars of knowledge utilisation.^18–26^ Instrumental use implies that a piece of research that presents BOD & VIE would directly influence a decision or act as a solution to a specific problem. Evidence use as such is systematic and a decision is traceable to a specific piece of research. Conceptual use of BOD & VIE follows a more complex process of knowledge translation, in which the research would indirectly impact the knowledge, understanding and/or attitude of the evidence receiver. This may lead to gradual changes in evidence receivers’ ways of thinking. Symbolic evidence use denotes research to be used more strategically. BOD & VIE would be drawn on to support a particular stance or to destabilise opposing positions. As such, BOD & VIE would be used anecdotally. Overall, evidence use models complement rather than contradict each other^27^ (table 1, online supplemental annex 1).

10.1136/bmjgh-2021-006827.supp1 Supplementary data

**Table 1:** Summary of the definitions of explanatory models to evidence use

**Instrumental**	
Instrumental evidence use describes the production and use of evidence in a linear process. This type of evidence use assumes the most rational approach.^63^ Scientific evidence is assumed to be instructive to the decision such that the impact is concrete and visible.^64^ Evidence use falls under the instrumental use category, when decisions are based on evaluation results.^63^	
*Knowledge-driven*	*Problem-solving*
Evidence leads policy and decision-making. New technologies or innovations that result from scientific evidence are adopted by decision-makers. Evidence producers ‘push’ their evidence towards the decision-maker. As a result, new policies emerge.	Evidence aims to reduce the decision-maker’s uncertainty around a specific problem by suggesting a specific solution.^64^ Decision-makers consult evidence when needed (research antedates the problem), or evidence producers are commissioned by evidence consumers to find solutions to problems.^64^ The policy goal is predetermined.^64^
**Conceptual**	
Evidence influences actions, but in less specific, more indirect ways. Research evidence permeates the action generation.	
*Enlightenment*	*Interactive*
Evidence use happens incrementally, leading to changes in the status quo in light of political realities as well as other factors and sources of evidence. Evidence may be used indirectly.^64^	An advancement of the enlightenment model, giving rise to disorderly interconnections, networks and relationships among stakeholders to enable the understanding of different paradigms and viewpoints. Continuous back-and-forth among stakeholders.^64^ Recognition of decision-making processes being complicated as stakeholders have different experiences with the evidence and diverging political insights, pressures and judgements.^46^
**Symbolic**	
Evidence is used to legitimise and support existing positions of evidence users. Evidence is used as ammunition to support predetermined positions or to delay decisions strategically.	
*Tactical*	*Political*
The evidence is not necessarily used as intended by its producer.^17^ The fact that evidence is being produced is used as a supporting argument by organisations for their responsiveness towards an issue.	The evidence consumer is uncontested and predetermined.^65^ Evidence is used to support the decision-maker’s standpoint.

Adapted from Davies *et al*^66^ and Weiss^17^ and complemented with accounts from later publications.

BOD & VIE, Burden of disease and vaccine impact estimates; GHO, Global health organization.

We conducted in-depth, semistructured interviews to explore how global BOD & VIE are used by GHOs as recommended by Hanney *et al* to study research utilisation.^28^

Beyer and Trice identify two main stakeholder groups in research utilisation: *evidence producers* and *evidence consumers*. For the purpose of this study, mathematical modellers commissioned to produce global BOD & VIE take up the role of *evidence producers. Producers* include individuals who work as epidemiologists or health economists at a research institution and who have extensive experience in the mathematical modelling of vaccine-preventable diseases. Moreover, they produce and disseminate BOD & VIE as academic outputs with the intent to inform decision-makers. *Producers* were initially identified as individuals, who are specialised in the mathematical modelling of a vaccine-preventable disease and were commissioned to produce the specific type of research evidence, global BOD & VIE by GHOs. *Evidence consumers* are employees at GHOs that commission research to, among other things, inform global resource allocation decisions and strengthen global immunisation systems through their organisational strategy. *Consumers* were initially identified by researching profiles of individuals employed at GHOs that grant funding to evidence producers and provide DAH to countries to support global vaccination initiatives. Evidence consumers do not have to be the final decision-makers but can sit along the pathway from receiving evidence to disseminating it to higher level decision-makers within the organisation (eg, board members taking organisation-wide decisions). This is important to note as previous studies have found that decisions are rarely made by a single individual^29 30^ and individuals often do not regard themselves as ultimate decision-makers.^31^

We acknowledge that the line between evidence producers and consumers is blurred.^32^ Often, *consumers* may have taken the role of *producers* in previous occupations or may even be *evidence producers* as part of their current position within their organisation. Therefore, we assign the informant to the organisation where they are employed at the time of the interview. We recognise that BOD & VIE may also be produced within the organisations at which the evidence consumers are employed. For this reason, we specifically asked informants about the use of global BOD & VIE that were formally commissioned to external constituencies.

Data were collected from purposively sampled individuals between March and September 2020 by PC. Snowball sampling was used to expand the study population to those who had not been identified previously but matched the outlined study eligibility criteria. Data saturation was achieved when ideas and/or opinions were repeated in different interviews. All interviews were conducted via the telecommuting software Zoom. Informed consent was obtained for every participant. A prewritten interview guide with open-ended questions was used (online supplemental annex 2). Additional questions were asked to clarify any information or to go into further depth on a topic. PC held the position as informed ‘outsider’, which allowed to gain trust of and achieve rapport with study participants (online supplemental annex 3). Interviews were audiotaped from which intelligent verbatim transcripts were produced. All study participants’ identities were pseudonymised to ensure confidentiality. Storage, organisation, searching and coding of transcripts were performed using NVivo V.12 Pro.^33^

10.1136/bmjgh-2021-006827.supp2 Supplementary data

10.1136/bmjgh-2021-006827.supp3 Supplementary data

In total, we approached 21 potential participants, 2 individuals refused to participate and 6 individuals did not respond. Interviewees included seven evidence producers (epidemiologists (n=6), a health economist (n=1)) and six evidence consumers (senior programme officers (n=4), team lead of the department of immunisation (n=1), medical epidemiologist in the department of immunisation (n=1)). All evidence producers held a position as postdoctoral fellow (n=3), assistant professor (n=2) or professor (n=2). Evidence consumers held a PhD (n=4), MSc (n=1), MPH (n=1) and/or MD (n=2). All interviewees were based in Europe (n=5) or North America (n=7), except for one evidence producer based in Asia.

### Analysis

The data were analysed using the framework method developed by Ritchie and Lewis,^34^ allowing for the comparison of predefined study population groups.^35^ Emergent themes were identified through initial coding by the lead author, PC. The preliminary codebook was reviewed by the second author, LC. Next, initial coding was succeeded by focused, selective coding borrowing concepts from the theoretical framework following a deductive approach. This was spot-checked by the second author, LC. The information was aggregated by theme and subtheme with respect to the two predefined study population groups. The approach allows to explore the specific use cases of evidence, while leaving space to discover unexpected or further emerging themes and relate findings to organisational theories and processes.

Our results focus on how global BOD & VIE are used and what actions are generated by GHOs as a result of receiving this evidence, with respect to the research utilisation spectrum and associated explanatory models (table 1, online supplemental annex 1), and draw on key themes identified in the analysis.

### Patient and public involvement

The design and conduct of the study, the development of the research question and outcome measures did not involve patients.

## Results

### Instrumental evidence use

Instrumental evidence use describes the systematic production and use of evidence in a linear process, implying that BOD & VIE would directly influence a decision or act as a solution to a specific problem. In line with this interpretation, there was general agreement and criticism among evidence producers and consumers that the production of BOD & VIE is dictated by evidence consumers, who set out the research agenda explicitly by providing funding for specific types of evidence. Yet, no specific decisions made by GHOs could be traced to a specific piece of research.

### Knowledge-driven model

For this model to apply, production of research evidence considered for use by evidence consumers should be driven by academic curiosity. Evidence producers and consumers agreed that this is rarely the case; however, opportunities exist for more of the processes along the evidence use route to be more knowledge driven.

#### Setting the research agenda

An evidence producer highlighted that evidence producers should lead the discussion on ‘what should be done’ that would be of value to evidence consumers. Thereby, the research agenda should be agreed on for each pathogen and for mathematical models of the same pathogen (ie, when having two or more mathematical models for one disease) (P1). The interviewee described the research agenda to be determined by evidence consumers:

We aren’t answering any questions about really what is actually happening at the policy level whether it is country-level policy and decision-making or global […] level decision-making. […], we are handed the demographics, we are handed the coverage assumptions and it is just running it through a population [pathogen] model, by age and time, to say what the impact of those assumptions would be. We are not really doing anything in terms of the research agenda. (P1)

#### Changes in evidence production

Evidence consumers and producers argued against the value of following the knowledge-driven route to evidence use. Informants stressed that changes in long-standing mathematical models can be a barrier to evidence use, as one evidence producer highlighted:

… for the most part, we are always a little cautious about dramatic changes in models because we’re really trying to translate observed changes in coverage, which is what [a GHO] has been investing in and understanding what that means for deaths averted [rather than] new modelling assumption. (C1)

Furthermore, evidence producers (P2, P7) referred to the delicate balancing act of responding to specific commissions for prescribed global burden models, while also still trying to fund models driven by more personal academic interests linked to knowledge gaps and methodological advances:

It’s sort of a little like a chicken and egg thing, I think. Like, you want to have those discussions, but […] I think, people don’t feel that they should give you the time or why would they give you the time if you haven’t done some work already? (P2)

There was a general agreement that a linear evidence production and consumption process, initiated by evidence producers, is rare and unrealistic given its reluctance towards innovation and changes in evidence.

### Problem-solving model

Interviewees addressed some principles of instrumental evidence use following the problem-solving model indirectly through the process of commissioning evidence producers and setting out deliverables through contractual agreements.

#### Evidence use through the commissioning of research

Both evidence producers and consumers stressed that evidence production is led by evidence consumers as they are commissioning the evidence production. Thereby, the evidence consumer is viewed as a major influence on evidence production and determines its direction and organisation (C4). They predetermine research questions and become ‘technical agencies’ determining the research agenda, as one interviewee stated:

When you, as an academic institute get funding, you will not argue with your donors, which are funding you. So, you follow the donor, you follow the money. […] [The funding organizations] are not only funding, they are doing more and more technical assistance, they are very much involved in the model set up, in everything from contracting to implementing. (C4)

An evidence producer also raised concern about the fact that the research agenda and problem definition is led and decided on by evidence consumers. While progress, goals and activities are discussed, grant deliverables that define the research agenda are set ex ante (P7). Evidence producers and consumers stated that this ignores the expertise of the evidence producer, who is arguably more aware of what requires further research (P2, C4). However, an evidence producer highlighted that commissioned research should be the result of discussions for the evidence to be ‘usable’ to decision-makers:

I’d say that the questions need to be applicable, usable, and so [the decision-makers] very much should be in the discussion of setting those questions. (P6)

It is evident that the interviewee sees value in more interaction between actors, also reflected in the interactive model (section *Interative Model*).

#### Problem definition

Questions posed by decision-makers when commissioning research may not necessarily be the ‘right’ questions or lead to decisions that are ‘right’ (P2). An evidence producer raised concerns that aggregating the impact of all mathematical models and projecting results to a global scale limits sufficient attention on specific national and subnational questions. For example, assumptions that all children who have received a first vaccine dose are also administered the second vaccine dose if required can lead to misleading outcomes (P1).

### Conceptual evidence use

In this next subsection, we explore the extent evidence permeates actions in more indirect ways leading to incremental changes in knowledge, understanding and/or attitude of evidence consumers as a result of using BOD & VIE.

### Enlightenment model

According to informants, evidence influences the organisational agenda incrementally over time. Which vaccines drive the most impact is ingrained in people’s knowledge as estimates have been seen many times (C2):

Our management isn’t using the numbers in a one-to-one way to make trade-offs. But it’s more that this is really foundational information for us to understand what the most important areas for us are to invest in. And then that drives more on an underlying fundamental basis, what we invest in immunizations. (C2)

Another evidence consumer highlighted that the evidence is not used by decision-makers at a high level to understand details of the impact of pathogen-specific interventions that are modelled mathematically (eg, which age groups are affected or the specific immunity gaps within countries) (C5).

Further, investment decisions made by an organisation’s management affect the work of all other departments within the organisation (C2):

So [BOD & VIE are] not the only thing that we would factor in, but it would be something that helps us to prioritize which countries we will most focus on investing in. And then that trickles down to all the rest of our work. (C2)

Therefore, research evidence shows to be ‘creeping’ into decisions, which is consistent with principles of the enlightenment model to evidence use. The applicability of the enlightenment model is further discussed under specific subthemes.

#### Argument-generating evidence

A subtheme applied to the enlightenment model to describe evidence use is that evidence has often been argument generating. The interviews conducted for this study highlighted several argument-generating questions oftentimes asked by evidence consumers after receiving BOD & VIE. Recurring questions were extracted from the interview data (box 1). The argument-generating component in the evidence use route suggests that social interaction is a sine qua non to facilitate evidence use for action generation (further discussed in section *Interactive Model*). If the evidence does not support the evidence consumer’s belief, and questions (eg, those in box 1) remain unanswered, the evidence consumer believes that the evidence is the problem (C6).

Box 1 **Argument-generating questions oftentimes posed by evidence consumers after receiving burden of disease and vaccine impact estimates (BOD & VIE)**

- What are the mathematical model differences in comparison to the previous year or reporting period? (C3)
- How well has the model done at predicting things that we know have happened in the past? Why are model estimates not meeting the consumers’ expectations? Can estimates be trusted? (P4)
- What explains changes in the mathematical model which affect the estimated impact? (C3, C1)
- Do we believe that these are good changes or are they going to end up going the other direction the next year when the modellers update their assumptions again? (C1)
- Is a change in BOD & VIE the result of something that happened in the world? Did the coverage decrease? Did the production not happen? Did something in the model change? Did the understanding of the epidemiology of a pathogen evolve? (C2)
- What drives differences in impact estimates from different models? (C3, C2)
- Are the multiple models valid for the same pathogen? Is there an error in one of the models? (C2)
- What can models do and cannot do? What improvements may be needed? With the aim of eliminating specific diseases, how does the mathematical model need to be aligned or changed? (P1)
- What is the population-level impact that goes beyond direct protection (herd immunity effect) in lower income settings where data are limited? (P5)
- How can estimates be additive in terms of not just using them in a vacuum, but using them to better understand and validate admin data estimates that come out? (C5)
- How do you interpret the relative contribution of different source of vaccination to the overall burden? That is, routine immunisation programmes (multiple doses), pulse national campaigns or national or subnational campaigns, outbreak response. (P3)
- What are the assumptions around the treatment for a disease? (C1)

As of interviewees’ accounts, the dissemination of BOD & VIE is often formally set out through reporting channels. This challenges the applicability of the enlightenment model, which defines the route of evidence permeation to be unguided and indirect. Tools to disseminate evidence suggest a guided evidence use process; however, the understanding of evidence cannot always be facilitated through these means as the argument-generating questions suggest.

### Interactive model

#### Relationships among stakeholders

Social interaction allows evidence to be produced with reference to what evidence consumers need to know in an iterative process (C4). Therefore, evidence consumers should be included in the early stages of the evidence development, including the set-up of the model structure and defining the outcomes that are required (C4, C5). Further, social interactions are required to answer the questions (eg, those in box 1).

Evidence producers and consumers have highlighted that interactions are required to ensure understanding of what can be researched, inherent limitations and caveats of evidence and to allow for evidence consumers to make improvement suggestions (P1).

Moreover, interactions through long-standing relationships reduce disruptiveness when the evidence changes over time, especially if conclusions are different from previous ones, leading to a shift in resource allocations (P7, P3). Changes in mathematical models and evidence supplied to consumers were viewed as a major point for discussion in interactions between evidence producers and consumers. Foundational knowledge, which has entrenched within evidence consumers over time, is being challenged, if new evidence does not meet its consumers’ expectations (P4). A lack of relationships may lead to challenges when trying to get the buy-in from stakeholders to use the evidence (P7, P2).

Overall, there was a consensus among evidence producers and consumers that the interactive model should define the route to evidence use. However, this model cannot necessarily be used to explain the current evidence use at stakeholders’ organisations. Interactions, especially at an early stage of evidence production, are required specifically for ensuring that evidence and its limitations and caveats are understood to promote its use and avoid evidence consumers ignoring it.

### Symbolic evidence use

Under the symbolic evidence use category, which stands in stark contrast with instrumental evidence use, evidence is used in a strategic manner for decisions that have already been taken. The symbolic use identified in this study aligns with the political explanatory model, that is, evidence is used to support the evidence user’s standpoint. No respondents spoke of the tactical model where solely the fact that research is being conducted is used in evidence users’ actions.

### Political model

An evidence consumer interviewee refers to ‘meta-questions’, which should be addressed by evidence producers (C5):

But [evidence producers are] answering kind of a more existential question rather than a specific programmatic question. (C5)

Probing revealed that evidence from mathematical models is mainly used for strategic thinking, planning purposes (C3), accountability and advocacy purposes when engaging with external funding partners of evidence consumers (C1, P6). BOD & VIE are used to solicit funding for further rounds of funding from donor partners (C1):

The impact numbers are probably the most important numbers that we use for replenishment efforts and for reporting to donors, so for high-level kind of accountability. (C1)

Sometimes, evidence is used for framing the setting or as contextual information for anyone trying to make the case for why the organisation should invest in a particular project over another one (C2). Evidence on vaccine impact comes into play when someone needs supporting evidence for his/her argument (C2) or to track the organisations’ progress (C5).

An evidence producer stated that despite estimates from multiple models for the same pathogen being hugely different, the main conclusions remain (P4). Details on BOD & VIE are of less interest to evidence consumers (C4). Rather, evidence consumers are interested in the direction of impact to support his/her argument (C4).

Overall, the political model describes evidence use once the evidence is engrained in the decision-makers’ understanding and the organisation’s agenda. Evidence consumers agree that research evidence is often used to inform and communicate targets to donors and to support an argument when wanting to receive more funding. Evidence producers did not provide any information alluding to the applicability of the political model.

## Discussion

This paper set out to examine how one type of commissioned research evidence (global BOD & VIE) is used by GHOs that aim to strengthen global immunisation systems. The findings highlight that BOD & VIE are not directly translated into GHOs’ actions, that is, systematically translated into vaccine resource allocation decisions, but are used to demonstrate accountability and solicit funding from external partners. While GHOs’ actions are partially influenced by BOD & VIE, the evidence is mainly used anecdotally. With regard to the conceptual framework used to analyse the results, the conceptual model and the political model best describe how evidence is used by GHOs.

Previous studies have furthered our understanding of evidence use by individuals, national governments and organisations on a variety of public health policies and decisions, such as maternal and child health^36^ and drug policies.^37^ However, to our knowledge, this is the first study to focus on the use of global epidemiological and economic research commissioned by GHOs using an organisational science perspective.

GHOs operate in a highly competitive environment.^38 39^ While international organisations receive support based primarily on the extent to which they can fulfil their mandate, philanthropic organisations’ funds are not dependent on support from donor partners.^40^ In this sense, the use of BOD & VIE is threefold. First, BOD & VIE are used to validate GHOs’ short-term funding objectives by comparing estimates with coverage data. Second, the evidence is used for strategically planning organisational mid-term to long-term actions by reflecting on the potential global health impact of their investments. Third, BOD & VIE are used to garner financial support from donor partners to pursue these objectives. These donor partners formally assess the impact of their budget allocation to international organisations through initiatives such as the Multilateral Organizations Performance Assessment Network.^38^ This explains GHOs’ high financial commitment towards commissioning research evidence in the form of global BOD & VIE, which demonstrates and markets the organisations’ impact, thus helping secure buy-in from their donor partners.

BOD & VIE also come into play when someone requires supporting evidence for their argument within the organisation. GHOs are ‘political’ organisations, in which several potentially conflicting interests and stakeholder groups exist.^38^ For this reason, BOD & VIE are used to promote a division’s cause internally. For example, BOD & VIE have been used to support internal decisions on why to invest in one vaccine over another.

Research evidence use by GHOs happens over a prolonged time in which many people within the organisation learn about the evidence. Over time, evidence on disease burden and vaccine impact becomes ingrained in people’s knowledge. From organisational theory, we learn that organisational norms and objectives are entrenched in employees’ minds.^41 42^ Actions are admixtures of scientific evidence and non-rational premises.^43^ Organisations’ actions embrace the ideas of their founders. Stinchcombe highlights that values are often preserved, and interests are protected by those who possess and retain power.^44^ With respect to this explicit role of agency and power within GHOs, BOD & VIE compete with other factors influencing organisational decisions. We found that organisational actions follow preset pathways established by executives’ opinions, interests and organisational norms rather than BOD & VIE forging new paths. As a result, we suggest further research into whether and how GHOs benefit from the accumulated knowledge, experiences and learnings of their employees. Further empirical research on organisational learning processes as suggested by Langley^45^ was beyond the remit of this research, in part to protect respondents’ confidentiality.

This study showed that, unsurprisingly, open interactions between evidence consumers and producers are vital to facilitate understanding of the evidence and its caveats and limitations.^46–48^ We found that evidence consumers’ questions, which set the research agenda, are not necessarily preview to or shaped by evidence producers. Previous studies suggest that evidence consumers, who participate in earlier phases of research, react favourably to results.^49^ However, rather than evidence consumers prescribing the research agenda, we found agreement among respondents that the problem definition should result from dialogues between evidence producers and consumers. To facilitate these dialogues and to enable the reflection of societal concerns, Beran *et al* advocate for platforms for exchange and the institutionalisation of a multistakeholder approach.^50^

Consistent with findings of previous studies, our study showed that trusted relationships can lead to trust in evidence and the acceptance and adoption of new evidence, which may otherwise be regarded as controversial or ‘negative’ evidence.^51–53^ This may be the case if foundational knowledge, which has entrenched within evidence consumers over time, is being challenged, not meeting their expectations. Early organisational studies showed that ‘negative’ research evidence can actively threaten the beliefs and worldviews of evidence consumers.^15 54^ All evidence consumers interviewed for this study had extensive prior education in infectious disease epidemiology, yet they were still reluctant to use findings from commissioned BOD & VIE, if these deviated too far from previous BOD & VIE or strongly held beliefs. Therefore, our study shows that organisational norms, objectives and/or other factors prevail direct, demonstrable use of evidence in this context.

While the benefits of interactions among evidence producers and consumers became obvious through this and other research, this process can also jeopardise the objectivity of the scientific community. The objectivity of the scientific community may be questioned if the political interests of research commissioners inhibit knowledge production. While this was not voiced by evidence consumers or producers interviewed for the purpose of this study, evidence-informed decision-making interventions that aim to enhance the dialogue between stakeholders need to reflect on the role of power and politics in the process of knowledge production.^55^

Mathematical models of infectious diseases have increasingly gained attractiveness among global health actors as they complement other epidemiological studies (eg, clinical trials^56^) and are less cost-intensive. They allow testing scenarios relatively quickly, while not being prone to the same ethical concerns. Despite inherent caveats and limitations of mathematical models, they provide high-level perspectives on global matters. As with other types of evidence, BOD & VIE should always be presented with underlying uncertainties and caveats.^57^ Our study suggests both producers and consumers share an honest assessment and understanding of the underlying uncertainties and caveats of mathematical models. Yet, when communicating results to external constituencies (eg, donor partners), point estimates are used to simplify messages and the important aspect of uncertainty is lost. This further supports our conclusion that evidence is used anecdotally when mobilising resources from donor partners, thereby serving a strategic purpose.^58^

Despite the growing number of organisations and actors in the global health system, it is strongly influenced by only a few entities that are headquartered in high-income countries,^59^ the Global North. As this study shows, mathematical models are predominantly used to demonstrate accountability by quantifying their impact and to proof organisations’ legitimacy to solicit funding from external partners. We acknowledge that the focus of this study has been a specific type of knowledge output commissioned. Other types of research, which are more explorative, innovative and less prescribed, and evidence produced by researchers from the Global South contribute differently to organisational actions taken. What the portfolio of evidence drawn on by GHOs embraces and how evidence use compares across this portfolio should be subject for further research. Moreover, this study focused on the use of evidence by philanthropic organisations or organisations with procurement mandates. Thus, the findings may not be fully transferable to the utilisation of BOD & VIE by advisory GHOs (eg, WHO), where such evidence may be used to give strategic and rapid guidance.

### Limitations

This study has its limitations. First, study participants’ responses may have been subject to social desirability. Both parties may have voiced their idealistic evidence use rather than the actual evidence use. Second, interviews were meant to have been conducted in person. Sometimes, it was difficult to grasp participants’ voices, opinions and non-verbal communication via video calls. However, this method of collecting primary data is considered a viable alternative to conducting interviews face to face.^60^ Third, the study relied on a small sample size and only reflected the view of a small proportion of employees of GHOs that commission evidence and researchers. However, given the highly concentrated nature of stakeholders operating at this global level of vaccine resource allocation, findings are likely to be qualitatively robust with regard to the sampled institutions. Lastly, all evidence producers and consumers were affiliated with an institution in a high-income country. Findings of this study may not reflect how evidence produced by evidence producers affiliated with academic institutions in LMICs is used by GHOs based in LMICs or local government bodies. We suggest this to be subject for further research. While our sample is a reflection of those most involved in this type of evidence production and consumption, it speaks to the perils of the ‘foreign gaze’ and ‘entrenched power asymmetries in global health partnerships’ that are being increasingly challenged by many of our respondents and the wider literature.^61 62^

## Conclusion

In conclusion, we found GHOs use BOD & VIE to demonstrate impact and accountability at the global level, often to solicit funding to support global immunisation efforts. GHOs that commission research evidence production and consider using research evidence should be engaged continuously throughout the research production process to maximise the value of BOD & VIE. Similarly, evidence producers should be engaged when GHOs use their research products. BOD & VIE are used to convince others of organisational legitimacy and may have had a direct influence on national, subnational and local vaccine resource allocation decisions. Yet, global BOD & VIE have been underused and only impacted GHOs’ actions indirectly. Arguably, global BOD & VIE have greater potential and can lead to more informed organisational actions, such as guiding vaccine resource allocation decisions across countries, not only to track progress and measure organisational success.

## Data Availability

No data are available. Interview transcripts cannot be shared to ensure confidentiality.
