# Supplementary material for: How are mathematical models and results from mathematical models of vaccine-preventable diseases used, or not, by global health organisations?
Source: BMJ Glob Health. 2021 Sep 6;6(9):e006827. doi: 10.1136/bmjgh-2021-006827 (PMC8422320; doi:10.1136/bmjgh-2021-006827)
Supplement: Supplementary data [file bmjgh-2021-006827supp001.pdf]

Annex 1

How are mathematical models and results from mathematical models of vaccine-preventable diseases used, or not, by global health organizations?

Theoretical orientation

Our analytical approach draws on key concepts of knowledge utilization. Given the vast number of studies, we only provide a brief account of the key theoretical and empirical findings and concepts relevant to this study and which underpin our assumptions.

Research utilization, a term used interchangeably with knowledge translation and research uptake has been studied in various disciplines. The individual decision-making process (i.e. at the micro level) has been mostly tackled by research psychologists (e.g. (1)), while decisions taken concerning national and international policies (i.e. the macro level) have been studied by political scientists (e.g. (2)). Finally, research utilization by organizations (i.e. the meso-level) has largely been the concern of organization and management scientists (3).

Research utilization by organizations aligns with the study of evidence-based management (EBM). EBM is derived from the model of evidence-based medicine and evidence-informed policymaking (4). A rich pool of literature has emerged around the use of evidence in different health settings over the past three decades. Existing studies predominantly focus on evidence use by physicians (5), managers of healthcare facilities and decision-makers who are directly involved in policymaking (6).

The framework presents findings from empirical research on a spectrum of research utilization typologies and relates these findings to organizational processes. Beyer and Trice's framework encompasses four organizational processes: information processing, affective bonding, strategy formulation and control, and action generation. *Information processing* concerns the cognition of research through deliberate actions of sensing, searching and diffusing evidence. *Affective bonding* refers to evidence consumers' feelings about research. Evidence consumers' affective reaction, receptivity and commitment towards evidence use shape this organizational process. The organizational process of *strategy formulation and control* addresses behaviours following the recognition of research evidence by selecting, evaluating and providing feedback on research evidence. *Action generation* pertains to the adoption, use, and, sometimes, the institutionalization of evidence (7). In previous analyses, scholars have set their focus on one of these organizational processes.

Instrumental evidence use follows a rational, prescriptive and systematic approach (8). Research is directly used in direct and specific ways (9). Either, questions identified by an organization lead to the commissioning of research to find a solution (problem-solving/engineering/policy-driven model) (10), or, research initiates organizational actions (classic/purist/knowledge-driven model) (9,11). The latter model is inherent to the assumption that stakeholders have a consensus on a goal and that evidence is sought and used actively in policy and decision-making processes (12).

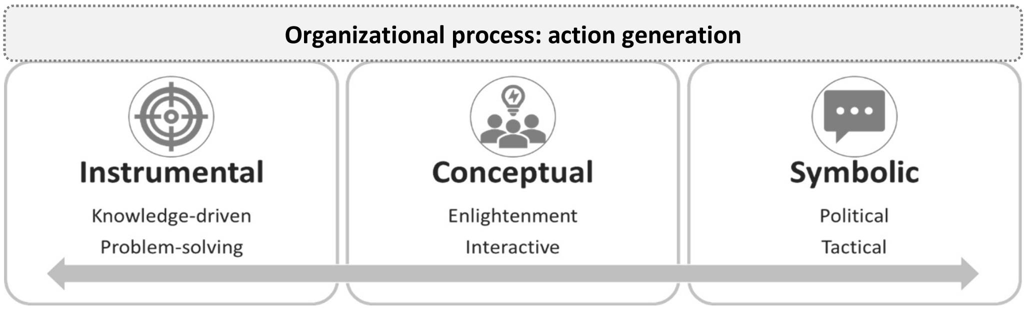

Figure 1 Spectrum of knowledge utilization and associated explanatory models as conceptualized by Weiss (56,57)

Annex 1

How are mathematical models and results from mathematical models of vaccine-preventable diseases used, or not, by global health organizations?

Within the category of conceptual evidence use, potential evidence users consider multiple sources of evidence and other contextual factors influence actions (9). This reflects the complexity of organizational processes arguably more realistically (13). Evidence production and consumption are therefore not bound to a linear, but a dynamic process. Conceptual evidence use can be described with the enlightenment (9) or the interactive model (11). In the enlightenment model evidence 'creeps' into decisions, shaping them incrementally over a longer period (14). The interactive model in this category of evidence use is defined similarly to the enlightenment model. Though, this model focuses more specifically on the existence of collaborations among stakeholders (12).

Lastly, symbolic evidence use can be further described through the political model or the tactical model (11). In the political model, evidence is used as a supportive device for a position that has already been taken. Organizations use evidence selectively and strategically. In the tactical model, the content of the evidence is negligible as the fact that research is being conducted in the field is used as an argument. Assumptions, in-depth explanations, critics and examples of each of the explanatory models were further outlined by Case (13). Variations of the six explanatory models are described by Hanney *et al* (11).

Annex 1

How are mathematical models and results from mathematical models of vaccine-preventable diseases used, or not, by global health organizations?

References

1. Michie S, Johnston M, Abraham C, Lawton R, Parker D, Walker A, et al. Making psychological theory useful for implementing evidence based practice: a consensus approach. *Quality & Safety in Health Care*. [Online] 2005;14(1): 26–33. Available from: doi:10.1136/qshc.2004.011155

2. Parkhurst J, Ghilardi L, Webster J, Hoyt J, Hill J, Lynch CA. Understanding evidence use from a programmatic perspective: conceptual development and empirical insights from national malaria control programmes. *Evidence & Policy: A Journal of Research, Debate and Practice*. [Online] 2020; Available from: doi:10.1332/174426420X15967828803210 [Accessed: 12th February 2021]

3. Reay T, Berta W, Kohn MK. What's the Evidence on Evidence-Based Management? *Academy of Management Perspectives*. [Online] 2009;23(4): 5–18. Available from: doi:10.5465/amp.23.4.5

4. Morrell K. The Narrative of 'Evidence Based' Management: A Polemic. *Journal of Management Studies*. [Online] 2008;45(3): 613–635. Available from: doi:10.1111/j.1467-6486.2007.00755.x

5. Contreary K, Collins A, Rich EC. Barriers to evidence-based physician decision-making at the point of care: a narrative literature review. *Journal of Comparative Effectiveness Research*. [Online] 2017;6(1): 51–63. Available from: doi:10.2217/cer-2016-0043

6. Peirson L, Ciliska D, Dobbins M, Mowat D. Building capacity for evidence informed decision making in public health: a case study of organizational change. *BMC Public Health*. [Online] 2012;12(1): 137. Available from: doi:10.1186/1471-2458-12-137

7. Beyer JM, Trice HM. The Utilization Process: A Conceptual Framework and Synthesis of Empirical Findings. *Administrative Science Quarterly*. [Online] 1982;27(4): 591. Available from: doi:10.2307/2392533

8. Albaek E. Between knowledge and power: Utilization of social science in public policy making. *Policy Sciences*. [Online] 1995;28(1): 79–100. Available from: doi:10.1007/BF01000821

9. Walt G. How far does research influence policy? *The European Journal of Public Health*. [Online] 1994;4(4): 233–235. Available from: doi:10.1093/eurpub/4.4.233

10. Gilson L, Orgill M, Shroff ZC. *Health policy analysis reader: the politics of policy change in low- and middle-income countries*. [Online] Geneva: World Health Organization; 2018. Available from: https://www.who.int/alliance-hpsr/resources/publications/hpa-reader/en/ [Accessed: 27th October 2020]

11. Hanney SR, Gonzalez-Block MA, Buxton MJ, Kogan M. The utilisation of health research in policy-making: concepts, examples and methods of assessment. *Health Research Policy and Systems*. [Online] 2003;1(1): 2. Available from: doi:10.1186/1478-4505-1-2

12. Weiss CH. The Many Meanings of Research Utilization. *Public Administration Review*. [Online] 1979;39(5): 426. Available from: doi:10.2307/3109916

Annex 1

How are mathematical models and results from mathematical models of vaccine-preventable diseases used, or not, by global health organizations?

13. Case KK. *From evidence to practice: the use of mathematical models to inform HIV programme planning and policy decision making*. [Online] [Thesis] [London]: Imperial College London; 2016. Available from: https://spiral.imperial.ac.uk:8443/handle/10044/1/60855

14. Radaelli CM. The role of knowledge in the policy process. *Journal of European Public Policy*. [Online] 1995;2(2): 159–183. Available from: doi:10.1080/13501769508406981
