# Supplementary material for: How are mathematical models and results from mathematical models of vaccine-preventable diseases used, or not, by global health organisations?
Source: BMJ Glob Health. 2021 Sep 6;6(9):e006827. doi: 10.1136/bmjgh-2021-006827 (PMC8422320; doi:10.1136/bmjgh-2021-006827)
Supplement: Supplementary data [file bmjgh-2021-006827supp002.pdf]

Annex 2

How are mathematical models and results from mathematical models of vaccine-preventable diseases used, or not, by global health organizations?

Semi-structured interview questions

The interview guide was designed with reference to publicly available semi-structured interview guides from similar studies (28,74) as well as the theoretical framework by Beyer and Trice (**Error! Reference source not found.**).

The following questions will be chosen depending on the profession of the study participant.

- What is your involvement in vaccine resource allocation decisions?
- What are the responsibilities regarding the work that you are reporting to the VIMC or that you are producing for the VIMC?
- How do you think are your estimates being used to inform policy and decision makers regarding the allocation of resources?
- In the case of receiving diverging information on the same matter, how is the information used in your [model/decision]?
- Is there a measure of quality control of information or knowledge shared with you?
- What is your experience when engaging with policy and decision makers?
- How do questions posed by policy and decision makers impact the design of your mathematical model?
- What initiated you designing the mathematical model that has been used to inform the VIMC?
- Have you received feedback about your mathematical model from policy and decision makers? What was the feedback? Have you amended your work respectively? If so, who?
- Have you received feedback about your mathematical model from the VIMC? What was the feedback? Have you amended your work respectively? If so, who?
- How you are engaging with other individuals acting upon vaccine resource allocation decisions?
- What is the intensity of engagement between you and other stakeholders who are acting upon vaccine resource allocation decisions is? Who are other stakeholders, who you are working with?

Annex 2

How are mathematical models and results from mathematical models of vaccine-preventable diseases used, or not, by global health organizations?

- How do you aim to answer questions by policy and decision makers using mathematical models?
- How are model results and estimates explained to resource allocation decision makers?
- How are mathematical models scoped and tailored to specific questions by policy and decision makers?
