# Supplementary material for: How are mathematical models and results from mathematical models of vaccine-preventable diseases used, or not, by global health organisations?
Source: BMJ Glob Health. 2021 Sep 6;6(9):e006827. doi: 10.1136/bmjgh-2021-006827 (PMC8422320; doi:10.1136/bmjgh-2021-006827)
Supplement: Supplementary data [file bmjgh-2021-006827supp003.pdf]

Annex 3

How are mathematical models and results from mathematical models of vaccine-preventable diseases used, or not, by global health organizations?

Reflexivity statement

PC conducted all interviews and conceptualized this study. She held the position as an informed "outsider" (1) with a background in epidemiology, economics and management science. She was therefore familiar with the technical terminology of both the evidence producers and consumers. Additionally, this background provided PC with an awareness of potential areas of sensitivity when probing on internal organizational actions and processes.

However, the interviewer's experience in the mathematical modelling of infectious diseases as well as in the conducting of research to support policy- and decision-makers allowed her to easily establish rapport and gain the trust of interviewees. Both, evidence producers and consumers were receptive and cooperative throughout the data collection process, and expressed confidence that their reflections on the use of evidence by global health organizations would be accurately understood and represented by the interviewer. By holding a 'shared experience' position with evidence producers, the interviewer had the ability to ask relevant follow-up questions and diminish distance during interviews. Furthermore, this position allowed her to comprehend implied content when analyzing the data. This was evident in interviewees' expressions, such as "you know…" and leaving sentences unfinished, implying interviewees had prior knowledge of the researchers' background.

Additionally, the interviewer's background in management science allowed her to develop a codebook that was informed by the conceptual framework developed by Beyer and Trice (2). Her prior knowledge of organizations' cultures and mathematical modelling of vaccine-preventable diseases allowed the author to highlight sub-themes and dive into specific organizational processes as well as subordinate questions around BOD & VIE in the analysis. This benefited the data collection as well as analysis as it shaped the thematic scope of the research, complementing the themes of the conceptual framework.

Yet, the interviewer had not worked at a global health organization classified as a philanthropic organisation or organisations with procurement mandates. This led the researcher to ask clarifying questions that focused on specific processes that had not necessarily been shared on organizations' websites or been explained in the literature. This benefitted the data analysis as evidence consumers answered questions with a lot of detail.

The researchers are aware of the risks when research is conducted by an informed "outsider", such as imposing own beliefs and perceptions by the researcher and the possibility of projecting biases (3). The review of the semi-structured interview guide (Annex 2) as well as the codebook by the second author, LC, aimed to reduce the possibility of introducing these biases and ensured an objective data analysis as the perspectives of two researchers were triangulated. Similarly, the second author was engaged in the data analysis by critically reviewing whether statements by evidence producers are clear enough to someone with no prior knowledge of infectious disease modelling. In cases where the interview content was unclear or ambiguous due to the use of technical terminology or the assumption that the authors had full understanding of the topic, the interviewee was contacted to clarify the argument. Through this approach, the authors aimed to reduce the danger of participants withholding information they assumed to be obvious to the researchers (1).

References

1. Berger R. Now I see it, now I don't: researcher's position and reflexivity in qualitative research. *Qualitative Research*. [Online] 2015;15(2): 219–234. Available from: doi:10.1177/1468794112468475

Annex 3

How are mathematical models and results from mathematical models of vaccine-preventable diseases used, or not, by global health organizations?

2. Beyer JM, Trice HM. The Utilization Process: A Conceptual Framework and Synthesis of Empirical Findings. *Administrative Science Quarterly*. [Online] 1982;27(4): 591. Available from: doi:10.2307/2392533

3. Drake P. Grasping at methodological understanding: a cautionary tale from insider research. *International Journal of Research & Method in Education*. [Online] 2010;33(1): 85–99. Available from: doi:10.1080/17437271003597592
